# Supplementary material for: A Genome-Wide Association Study of Metabolic Syndrome in the Taiwanese Population
Source: Nutrients. 2023 Dec 25;16(1):77. doi: 10.3390/nu16010077 (PMC10780952; doi:10.3390/nu16010077)
Supplement: Supplementary file 1 [file nutrients-16-00077-s001.zip › nutrients-2734759-supplementary.pdf]

A genome-wide association study (GWAS) of metabolic syn-drome in the Taiwanese population

Supplementary Table S1. GWAS of Mets (Binary) disclosed 549 independent loci in our cohort with P < 5*10^{-8}

SNP	Chr	Position	Effect allele	Other allele	EAF	Beta coefficient	SE	P	Nereast gene
rs730497	7	44184122	G	A	0.207	0.084341	0.01306	9.14E-11	GCK
rs2908289	7	44184343	G	A	0.207	0.084341	0.01306	1.10E-10	GCK
rs2971670	7	44186502	C	T	0.2047	0.086178	0.01315	6.47E-11	GCK
rs1799884	7	44189469	C	T	0.205	0.08526	0.01312	6.94E-11	GCK
rs2971669	7	44192179	C	T	0.3654	0.062975	0.01093	8.10E-09	GCK
rs6975024	7	44192287	T	C	0.2145	0.077887	0.01289	1.81E-09	GCK
rs2908286	7	44195138	C	T	0.2081	0.087095	0.01305	3.02E-11	GCK
rs1004558	7	44200808	C	T	0.208	0.087095	0.01306	2.78E-11	YKT6
rs2971668	7	44203839	G	C	0.2168	0.080658	0.01285	4.15E-10	YKT6
rs2971667	7	44205461	T	C	0.2083	0.086178	0.01304	3.42E-11	YKT6
rs917793	7	44206254	A	T	0.2083	0.086178	0.01304	4.16E-11	YKT6
rs2908282	7	44209229	G	A	0.2082	0.087095	0.01305	3.05E-11	YKT6
rs3840674	7	44212591	CTG	C	0.2087	0.086178	0.01308	4.86E-11	YKT6
rs878521	7	44216044	G	A	0.3661	0.062035	0.01093	1.37E-08	YKT6, CAMK2B
rs12539351	7	73563782	A	G	0.08327	-0.10458	0.01883	2.75E-08	TBL2, BCL7B
rs1584033528	7	73574811	C	CA	0.08814	-0.10425	0.01836	1.35E-08	TBL2
rs35732917	7	73598939	T	C	0.09015	-0.09927	0.01817	4.68E-08	MLXIPL
rs13235543	7	73599571	C	T	0.09008	-0.09916	0.01818	4.86E-08	MLXIPL

rs35368205	7	73603327	C	T	0.08072	-0.11116	0.01908	5.62E-09	MLXIPL
rs61010704	7	73605645	A	G	0.07847	-0.12364	0.01931	1.54E-10	MLXIPL
rs3812316	7	73606007	C	G	0.07842	-0.12466	0.01931	1.06E-10	MLXIPL
rs6968170	7	73606346	G	A	0.07848	-0.12386	0.0193	1.36E-10	MLXIPL
rs13246993	7	73608416	G	A	0.07847	-0.12398	0.0193	1.32E-10	MLXIPL
rs12531645	7	73609551	G	A	0.07844	-0.12319	0.01931	1.74E-10	MLXIPL
rs35512732	7	73610432	TC	T	0.07847	-0.12409	0.0193	1.31E-10	MLXIPL
rs13234131	7	73611645	A	G	0.09162	-0.10781	0.01801	2.17E-09	MLXIPL
rs17145750	7	73612048	C	T	0.09203	-0.10703	0.01797	2.62E-09	MLXIPL
rs6460047	7	73628113	T	C	0.1016	-0.09861	0.01726	1.11E-08	VPS37D, MLXIPL
rs7805504	7	73628255	T	C	0.1013	-0.10004	0.01727	6.88E-09	VPS37D, MLXIPL
rs7786376	7	73628284	A	G	0.1026	-0.09883	0.0172	8.96E-09	VPS37D, MLXIPL
rs34958196	7	73628771	G	A	0.1315	-0.09135	0.01556	4.46E-09	VPS37D, MLXIPL
rs34430945	7	73629335	T	C	0.1311	-0.0908	0.01557	5.44E-09	VPS37D, MLXIPL
rs7784668	7	73631153	T	C	0.1304	-0.09376	0.01562	1.98E-09	VPS37D, MLXIPL
rs13234805	7	73634852	G	A	0.1276	-0.08982	0.01586	1.50E-08	MLXIPL
rs10091649	8	19898910	T	G	0.08561	-0.10825	0.01869	6.84E-09	LPL
rs10102717	8	19899302	C	T	0.08361	-0.10781	0.01888	1.14E-08	LPL
rs1441770	8	19899808	G	A	0.08334	-0.11116	0.01891	4.20E-09	INTS10, LPL
rs3988301	8	19912868	T	G	0.1435	-0.14491	0.01494	3.03E-22	LPL
rs28834434	8	19917379	C	A	0.1369	-0.14514	0.01509	6.75E-22	LPL
rs4466415	8	19919470	A	C	0.1478	-0.14191	0.01471	5.16E-22	LPL

rs77899453	8	19922799	G	A	0.1365	-0.14572	0.01514	6.27E-22	LPL
rs2119692	8	19922946	G	C	0.1374	-0.14549	0.0151	5.61E-22	LPL
rs139001474	8	19931094	T	A	0.1462	-0.14226	0.01474	4.83E-22	LPL
rs149764042	8	19931164	G	C	0.05929	-0.18452	0.02199	4.71E-17	LPL
rs4592053	8	19931255	A	G	0.1467	-0.14191	0.01472	5.52E-22	LPL
rs150331294	8	19932487	T	A	0.1419	-0.14445	0.01489	2.92E-22	LPL
rs73667472	8	19952184	T	C	0.05124	-0.21456	0.02353	7.70E-20	LPL
rs343	8	19953276	C	A	0.1423	-0.0931	0.01518	8.66E-10	LPL
rs249	8	19953495	T	C	0.05179	-0.21493	0.02339	4.10E-20	LPL
rs250	8	19953512	A	ATG	0.146	-0.09146	0.01503	1.19E-09	LPL
rs255	8	19954390	T	C	0.2011	-0.14191	0.01313	3.02E-27	LPL
rs1554517791	8	19956458	G	GT	0.198	-0.14618	0.01324	2.41E-28	LPL
rs305	8	19959890	A	G	0.184	-0.15993	0.01344	1.15E-32	LPL
rs308	8	19959965	T	G	0.06921	-0.20297	0.02034	1.88E-23	LPL
rs314	8	19960531	G	A	0.1849	-0.15935	0.01341	1.46E-32	LPL
rs325	8	19961817	T	C	0.08972	-0.20212	0.01806	4.23E-29	LPL
rs326	8	19961928	A	G	0.1852	-0.16134	0.01338	1.71E-33	LPL
rs328	8	19962213	C	G	0.08972	-0.20224	0.01806	3.97E-29	LPL
rs331	8	19962894	G	A	0.1839	-0.16146	0.01341	2.35E-33	LPL
rs12679834	8	19962922	T	C	0.08967	-0.20248	0.01806	3.51E-29	LPL
rs75278536	8	19963914	T	G	0.08947	-0.20199	0.01808	5.48E-29	LPL
rs146265243	8	19963956	GT	G	0.08948	-0.20212	0.01808	5.40E-29	LPL

rs77069344	8	19964271	T	G	0.08947	-0.20199	0.01808	5.55E-29	LPL
rs1803924	8	19966163	C	T	0.08965	-0.20199	0.01806	4.97E-29	LPL
rs3735964	8	19966534	C	A	0.0897	-0.20175	0.01806	5.45E-29	LPL
rs1059611	8	19967052	T	C	0.08973	-0.20175	0.01805	5.44E-29	LPL
rs10645926	8	19967115	C	CTT	0.0897	-0.20163	0.01806	6.00E-29	LPL
rs3916027	8	19967357	G	A	0.1815	-0.16111	0.01348	6.24E-33	LPL
rs77150190	8	19968791	G	C	0.08176	-0.20555	0.01883	1.01E-27	RPL30P9, LPL
rs77729186	8	19968807	A	G	0.08176	-0.20555	0.01883	1.01E-27	RPL30P9, LPL
rs79108221	8	19969190	C	T	0.08174	-0.20543	0.01884	1.10E-27	RPL30P9, LPL
rs1011685	8	19973258	C	T	0.08904	-0.19943	0.01812	3.71E-28	RPL30P9, LPL
rs10096633	8	19973410	C	T	0.08915	-0.20028	0.01812	2.10E-28	RPL30P9, LPL
rs17482753	8	19975135	G	T	0.0887	-0.19833	0.01816	9.00E-28	RPL30P9, LPL
rs78963197	8	19979135	T	C	0.08632	-0.19541	0.01844	2.97E-26	RPL30P9, LPL
rs74855321	8	19979473	C	T	0.08801	-0.19662	0.01823	4.19E-27	RPL30P9, LPL
rs17410914	8	19985951	C	A	0.08569	-0.20616	0.01845	5.51E-29	RPL30P9, LPL
rs117227752	8	19986350	G	A	0.08854	-0.20053	0.01817	2.45E-28	RPL30P9, LPL
rs59147390	8	19986368	T	C	0.08854	-0.20053	0.01817	2.49E-28	RPL30P9, LPL
rs12678919	8	19986711	A	G	0.0889	-0.19894	0.01813	5.14E-28	RPL30P9, LPL
rs7819706	8	19986904	A	G	0.08844	-0.20028	0.01818	3.30E-28	RPL30P9, LPL
rs115129770	8	19986928	C	G	0.08844	-0.20028	0.01818	3.30E-28	RPL30P9, LPL
rs149670831	8	19986936	CT	C	0.08844	-0.20028	0.01818	3.30E-28	RPL30P9, LPL
rs78458743	8	19988803	T	G	0.08843	-0.2015	0.01819	1.56E-28	RPL30P9, LPL

rs12682115	8	19989549	G	A	0.08843	-0.2015	0.01819	1.56E-28	RPL30P9, LPL
rs78013771	8	19989712	T	C	0.08843	-0.2015	0.01819	1.57E-28	RPL30P9, LPL
rs17410962	8	19990569	G	A	0.08852	-0.20089	0.01817	2.10E-28	RPL30P9, LPL
rs117604010	8	19990885	G	A	0.06833	-0.2015	0.02045	6.67E-23	RPL30P9, LPL
rs17489185	8	19990988	G	C	0.08852	-0.20089	0.01817	2.07E-28	RPL30P9, LPL
rs17410983	8	19991182	C	T	0.08852	-0.20089	0.01817	2.05E-28	RPL30P9, LPL
rs17489226	8	19991367	A	T	0.08852	-0.20089	0.01817	2.04E-28	RPL30P9, LPL
rs17410996	8	19991761	T	G	0.08851	-0.20004	0.01818	3.60E-28	RPL30P9, LPL
rs75801420	8	19992159	T	G	0.08863	-0.19906	0.01817	6.54E-28	RPL30P9, LPL
rs17091905	8	19992246	G	A	0.08854	-0.20138	0.01817	1.57E-28	RPL30P9, LPL
rs114964604	8	19992478	A	G	0.0878	-0.20065	0.01826	4.53E-28	RPL30P9, LPL
rs73600084	8	19992481	T	C	0.0878	-0.20065	0.01826	4.53E-28	RPL30P9, LPL
rs76975037	8	19993997	C	A	0.08848	-0.20102	0.01818	1.95E-28	RPL30P9, LPL
rs17489268	8	19994534	T	A	0.1811	-0.16076	0.01351	1.26E-32	RPL30P9, LPL
rs17411024	8	19994623	G	A	0.08833	-0.20065	0.01819	2.77E-28	RPL30P9, LPL
rs17091909	8	19994645	G	T	0.08844	-0.20114	0.01819	1.96E-28	RPL30P9, LPL
rs17411031	8	19994799	C	G	0.1812	-0.16087	0.01351	1.15E-32	RPL30P9, LPL
rs17489282	8	19995007	C	T	0.1812	-0.16064	0.01351	1.34E-32	RPL30P9, LPL
rs4922117	8	19995075	T	C	0.1812	-0.16076	0.01351	1.16E-32	RPL30P9, LPL
rs1822200	8	19995318	G	T	0.1812	-0.16076	0.01351	1.16E-32	RPL30P9, LPL
rs1372344	8	19995428	A	T	0.1812	-0.16076	0.01351	1.16E-32	RPL30P9, LPL
rs1992442	8	19995878	A	C	0.1814	-0.16017	0.01351	2.20E-32	RPL30P9, LPL

rs2410618	8	19995989	G	A	0.1811	-0.16134	0.01351	7.45E-33	RPL30P9, LPL
rs1992443	8	19996076	G	A	0.1811	-0.16134	0.01351	7.45E-33	RPL30P9, LPL
rs920588	8	19996756	C	T	0.1812	-0.16111	0.01351	8.56E-33	RPL30P9, LPL
rs920589	8	19996850	G	T	0.1811	-0.16123	0.01351	7.88E-33	RPL30P9, LPL
rs4425772	8	19996989	C	T	0.1806	-0.16193	0.01353	5.48E-33	RPL30P9, LPL
rs4628269	8	19996998	C	T	0.1806	-0.16193	0.01353	5.48E-33	RPL30P9, LPL
rs2410619	8	19997042	C	A	0.1811	-0.16123	0.01351	7.88E-33	RPL30P9, LPL
rs4320561	8	19997126	C	A	0.1811	-0.1604	0.01352	1.81E-32	RPL30P9, LPL
rs2410620	8	19997149	C	T	0.1811	-0.1604	0.01352	1.81E-32	RPL30P9, LPL
rs2410621	8	19997171	T	C	0.1811	-0.1604	0.01352	1.81E-32	RPL30P9, LPL
rs2410623	8	19997378	A	G	0.1812	-0.16111	0.01351	8.61E-33	RPL30P9, LPL
rs17411113	8	19997495	C	G	0.08851	-0.20163	0.01818	1.39E-28	RPL30P9, LPL
rs79734126	8	19997519	C	T	0.06787	-0.2004	0.02052	1.61E-22	RPL30P9, LPL
rs34564316	8	19997710	C	T	0.1812	-0.16111	0.01353	1.07E-32	RPL30P9, LPL
rs17411126	8	19997761	T	C	0.1812	-0.16123	0.01351	7.73E-33	RPL30P9, LPL
rs17411133	8	19997820	T	A	0.1812	-0.16111	0.01351	8.33E-33	RPL30P9, LPL
rs17489373	8	19997833	G	A	0.1812	-0.16111	0.01351	8.58E-33	RPL30P9, LPL
rs17411168	8	19998089	T	C	0.1815	-0.16029	0.01349	1.45E-32	RPL30P9, LPL
rs34932218	8	19998150	A	G	0.1816	-0.16064	0.01349	1.11E-32	RPL30P9, LPL
rs4523270	8	19999028	T	C	0.1816	-0.15993	0.01348	1.90E-32	RPL30P9, LPL
rs2103325	8	20000832	C	T	0.1812	-0.16123	0.01349	6.50E-33	RPL30P9, LPL
rs1581675	8	20000988	T	A	0.1812	-0.16134	0.01349	5.83E-33	RPL30P9, LPL

rs4490856	8	20001609	T	C	0.3361	-0.10425	0.01118	1.11E-20	RPL30P9, LPL
rs2119690	8	20002028	G	A	0.1813	-0.1617	0.01349	4.36E-33	RPL30P9, LPL
rs2165556	8	20002116	C	T	0.1813	-0.1617	0.01349	4.42E-33	RPL30P9, LPL
rs12541912	8	20002280	G	C	0.1817	-0.16087	0.01348	7.51E-33	RPL30P9, LPL
rs79236614	8	20002949	C	G	0.08838	-0.20114	0.01818	1.92E-28	RPL30P9, LPL
rs34818360	8	20003236	G	A	0.1811	-0.16123	0.01349	6.77E-33	RPL30P9, LPL
rs4083261	8	20003715	T	C	0.1817	-0.16099	0.01348	7.20E-33	RPL30P9, LPL
rs3844510	8	20003850	A	C	0.1814	-0.16134	0.01349	5.79E-33	RPL30P9, LPL
rs2410625	8	20004709	C	T	0.1813	-0.16158	0.01349	4.62E-33	RPL30P9, LPL
rs4644277	8	20005059	T	C	0.1813	-0.16158	0.01349	4.62E-33	RPL30P9, LPL
rs4389957	8	20005235	G	A	0.3374	-0.09971	0.01117	4.42E-19	RPL30P9, LPL
rs1441766	8	20005277	A	T	0.1832	-0.16005	0.01347	1.43E-32	RPL30P9, LPL
rs1441764	8	20005513	C	T	0.1817	-0.16099	0.01348	6.77E-33	RPL30P9, LPL
rs6586884	8	20005847	T	C	0.08846	-0.20138	0.01818	1.61E-28	RPL30P9, LPL
rs6999813	8	20005960	T	A	0.08846	-0.20138	0.01818	1.61E-28	RPL30P9, LPL
rs2119689	8	20005996	C	T	0.1813	-0.16158	0.01349	4.62E-33	RPL30P9, LPL
rs1398468722	8	20006328	G	GA	0.179	-0.16287	0.01358	3.88E-33	RPL30P9, LPL
rs1441763	8	20007045	T	C	0.1813	-0.16158	0.01349	4.62E-33	RPL30P9, LPL
rs1441762	8	20007176	G	A	0.1814	-0.16193	0.01349	3.64E-33	RPL30P9, LPL
rs1441761	8	20007237	G	C	0.1807	-0.16099	0.01351	9.85E-33	RPL30P9, LPL
rs1441760	8	20007238	C	T	0.1807	-0.16099	0.01351	9.85E-33	RPL30P9, LPL
rs2083637	8	20007664	A	G	0.1814	-0.16087	0.01349	8.73E-33	RPL30P9, LPL

rs2083636	8	20007752	T	G	0.1818	-0.16064	0.01347	9.00E-33	RPL30P9, LPL
rs77237194	8	20007944	A	T	0.08855	-0.2004	0.01817	2.82E-28	RPL30P9, LPL
rs35495249	8	20007998	C	A	0.1804	-0.16017	0.01353	2.51E-32	RPL30P9, LPL
rs894211	8	20008236	C	T	0.1805	-0.15993	0.01352	2.93E-32	RPL30P9, LPL
rs894210	8	20008332	G	A	0.3385	-0.10159	0.01115	7.75E-20	RPL30P9, LPL
rs765547	8	20008763	G	A	0.1815	-0.16064	0.01348	9.53E-33	RPL30P9, LPL
rs765548	8	20009083	C	T	0.1815	-0.16064	0.01348	1.01E-32	RPL30P9, LPL
rs765549	8	20009120	A	G	0.1818	-0.16017	0.01347	1.43E-32	RPL30P9, LPL
rs1441758	8	20009367	A	T	0.1815	-0.16064	0.01348	1.01E-32	RPL30P9, LPL
rs139547332	8	20009665	C	CT	0.06833	-0.20053	0.02045	1.06E-22	RPL30P9, LPL
rs9644637	8	20009709	T	C	0.1815	-0.16064	0.01348	1.01E-32	RPL30P9, LPL
rs9644638	8	20009889	C	T	0.1811	-0.16017	0.0135	1.76E-32	RPL30P9, LPL
rs11986942	8	20009934	C	G	0.1818	-0.16017	0.01347	1.43E-32	RPL30P9, LPL
rs34327087	8	20009983	G	T	0.1814	-0.16076	0.01349	8.97E-33	RPL30P9, LPL
rs34345068	8	20010025	G	T	0.1814	-0.16064	0.01349	1.00E-32	RPL30P9, LPL
rs2009493	8	20010342	G	A	0.1815	-0.16064	0.01348	9.92E-33	RPL30P9, LPL
rs1837843	8	20010591	C	T	0.1778	-0.16158	0.0136	1.38E-32	RPL30P9, LPL
rs1837842	8	20010779	T	C	0.1815	-0.16064	0.01348	9.92E-33	RPL30P9, LPL
rs1441757	8	20010866	G	C	0.1815	-0.16064	0.01348	9.92E-33	RPL30P9, LPL
rs1441755	8	20011377	T	C	0.1815	-0.16064	0.01348	9.92E-33	RPL30P9, LPL
rs1441754	8	20011428	G	A	0.1806	-0.16146	0.01352	6.48E-33	RPL30P9, LPL
rs4406409	8	20011460	T	C	0.1805	-0.16181	0.01353	5.52E-33	RPL30P9, LPL

rs1441753	8	20011553	G	A	0.1815	-0.16064	0.01348	9.92E-33	RPL30P9, LPL
rs4126104	8	20011855	G	T	0.1815	-0.16064	0.01348	9.92E-33	RPL30P9, LPL
rs35687633	8	20012476	A	G	0.1811	-0.1604	0.01351	1.71E-32	RPL30P9, LPL
rs34942551	8	20012504	T	G	0.1811	-0.1604	0.01351	1.70E-32	RPL30P9, LPL
rs34499461	8	20012516	G	C	0.181	-0.16017	0.01352	2.27E-32	RPL30P9, LPL
rs35369244	8	20012533	G	C	0.181	-0.16017	0.01352	2.10E-32	RPL30P9, LPL
rs35739466	8	20012576	G	A	0.1782	-0.16064	0.01362	4.27E-32	RPL30P9, LPL
rs35617716	8	20012752	T	A	0.1792	-0.15513	0.01356	2.81E-30	RPL30P9, LPL
rs35237252	8	20012760	C	A	0.1793	-0.15432	0.01356	5.29E-30	RPL30P9, LPL
rs78810414	8	20013602	C	T	0.07372	-0.1976	0.0198	1.91E-23	RPL30P9, LPL
rs1372343	8	20013809	C	T	0.1688	-0.15269	0.01391	5.03E-28	RPL30P9, LPL
rs1372342	8	20013820	T	A	0.1688	-0.1528	0.01391	4.82E-28	RPL30P9, LPL
rs1372341	8	20013821	T	A	0.1688	-0.1528	0.01391	4.82E-28	RPL30P9, LPL
rs1372340	8	20013861	G	A	0.1658	-0.15338	0.01402	7.28E-28	RPL30P9, LPL
rs2410627	8	20014002	T	C	0.1693	-0.1521	0.01389	7.20E-28	RPL30P9, LPL
rs7461115	8	20014029	C	G	0.1693	-0.1521	0.01389	6.65E-28	RPL30P9, LPL
rs2410628	8	20014118	C	T	0.1693	-0.15164	0.01389	1.01E-27	RPL30P9, LPL
rs2410629	8	20014126	T	C	0.1694	-0.15187	0.01389	7.76E-28	RPL30P9, LPL
rs2898495	8	20014381	A	G	0.1693	-0.1521	0.01389	6.52E-28	RPL30P9, LPL
rs4593558	8	20014497	A	G	0.1693	-0.15222	0.01389	5.79E-28	RPL30P9, LPL
rs6986010	8	20015269	T	C	0.1693	-0.1521	0.01389	6.29E-28	RPL30P9, LPL
rs35465966	8	20016045	C	A	0.1689	-0.15269	0.01391	4.87E-28	RPL30P9, LPL

rs34693282	8	20016055	A	G	0.1692	-0.15245	0.0139	5.26E-28	RPL30P9, LPL
rs17489539	8	20016071	T	A	0.1692	-0.15071	0.0139	2.02E-27	RPL30P9, LPL
rs4922118	8	20016634	C	T	0.1691	-0.15164	0.01392	1.25E-27	RPL30P9, LPL
rs4922119	8	20016642	C	T	0.1691	-0.15164	0.01392	1.25E-27	RPL30P9, LPL
rs4375019	8	20016987	T	A	0.1656	-0.1535	0.01402	6.83E-28	RPL30P9, LPL
rs141287949	8	20017421	AAGAC	A	0.07402	-0.19821	0.01974	1.04E-23	RPL30P9, LPL
rs28675909	8	20018723	A	T	0.0738	-0.2004	0.01976	3.60E-24	RPL30P9, LPL
rs79198716	8	20018753	C	T	0.07394	-0.20089	0.01974	2.62E-24	RPL30P9, LPL
rs78300069	8	20019013	C	T	0.07436	-0.20065	0.01969	2.15E-24	RPL30P9, LPL
rs76737188	8	20019083	G	C	0.07783	-0.19565	0.01927	3.32E-24	RPL30P9, LPL
rs7016880	8	20019235	G	C	0.07793	-0.19504	0.01926	4.29E-24	RPL30P9, LPL
rs7007609	8	20019286	T	C	0.07783	-0.19565	0.01927	3.32E-24	RPL30P9, LPL
rs7007797	8	20019459	T	G	0.07783	-0.19565	0.01927	3.30E-24	RPL30P9, LPL
rs7004149	8	20019697	A	G	0.07783	-0.19565	0.01927	3.30E-24	RPL30P9, LPL
rs7004158	8	20019722	A	G	0.07783	-0.19565	0.01927	3.30E-24	RPL30P9, LPL
rs6983430	8	20019742	G	C	0.07783	-0.19565	0.01927	3.30E-24	RPL30P9, LPL
rs77219697	8	20019950	T	C	0.07784	-0.19565	0.01927	3.23E-24	RPL30P9, LPL
rs12678603	8	20020221	C	T	0.07439	-0.20053	0.01969	2.33E-24	RPL30P9, LPL
rs12678604	8	20020262	C	G	0.07436	-0.20065	0.01969	2.13E-24	RPL30P9, LPL
rs112994260	8	20021193	T	A	0.07782	-0.19516	0.01928	4.31E-24	RPL30P9, LPL
rs142117848	8	20021359	C	A	0.07782	-0.19529	0.01928	4.15E-24	RPL30P9, LPL
rs75082673	8	20022298	G	A	0.07433	-0.2004	0.01969	2.50E-24	RPL30P9, LPL

rs11989309	8	20022819	C	T	0.07779	-0.19541	0.01928	3.93E-24	RPL30P9, LPL
rs75675187	8	20023215	C	T	0.07778	-0.19529	0.01928	4.23E-24	RPL30P9, LPL
rs74511360	8	20023529	G	A	0.0743	-0.20077	0.01969	2.14E-24	RPL30P9, LPL
rs78404258	8	20023547	A	G	0.07786	-0.19492	0.01927	4.89E-24	RPL30P9, LPL
rs74615535	8	20023725	G	A	0.07774	-0.19601	0.01929	2.86E-24	RPL30P9, LPL
rs60223219	8	20024290	A	G	0.07776	-0.19577	0.01928	3.27E-24	RPL30P9, LPL
rs7816447	8	20025439	T	C	0.07663	-0.19358	0.01944	2.32E-23	RPL30P9, LPL
rs114650503	8	20026746	G	A	0.0743	-0.20053	0.0197	2.40E-24	RPL30P9, LPL
rs9644639	8	20027436	C	G	0.07421	-0.20126	0.01971	1.82E-24	RPL30P9, LPL
rs11984636	8	20028215	T	C	0.07489	-0.19784	0.01963	6.84E-24	RPL30P9, LPL
rs4244457	8	20041535	C	T	0.3119	-0.10259	0.01129	1.05E-19	RPL30P9, LPL
rs7826306	8	20043160	G	C	0.3147	-0.09949	0.01129	1.25E-18	RPL30P9, LPL
rs74983646	8	20045739	G	A	0.08892	-0.1411	0.01832	1.34E-14	RPL30P9, LPL
rs11991231	8	20050401	T	C	0.1035	-0.14595	0.0171	1.37E-17	RPL30P9, LPL
rs115849089	8	20054859	G	A	0.1005	-0.14665	0.01732	2.56E-17	RPL30P9, LPL
rs4128744	8	20062144	C	T	0.1016	-0.14595	0.01724	2.61E-17	RPL30P9, LPL
rs9644568	8	20071071	G	A	0.1026	-0.14954	0.01713	2.64E-18	RPL30P9, LPL
rs1445190	8	34398333	A	G	0.4986	0.062035	0.01068	6.13E-09	RPL10AP3, LINC01288
rs2404558	8	34408317	T	A	0.4983	0.062975	0.01068	3.33E-09	RPL10AP3, LINC01288
rs4486200	8	34436234	C	T	0.4972	0.063913	0.0107	2.31E-09	RPL10AP3, LINC01288
rs2980888	8	125495066	C	T	0.2935	0.062975	0.01157	4.48E-08	TRIB1, LINC00861
rs2954038	8	125495147	A	C	0.2938	0.063913	0.01157	3.33E-08	TRIB1, LINC00861

rs10466351	11	92964815	C	T	0.4492	0.058269	0.01056	2.71E-08	MTNR1B, SNRPGP16
rs7941837	11	92966500	A	T	0.4503	0.058269	0.01059	3.14E-08	MTNR1B, SNRPGP16
rs10830963	11	92975544	C	G	0.443	0.059212	0.01058	1.80E-08	MTNR1B
rs10892001	11	116634723	G	A	0.1493	0.086178	0.01517	1.46E-08	LOC107984372
rs10892002	11	116634775	G	C	0.1491	0.086178	0.01518	1.22E-08	LOC107984372
rs513533	11	116648641	A	G	0.1231	0.090754	0.01613	2.04E-08	LINC02702
rs516043	11	116648867	C	G	0.1231	0.089841	0.01613	2.89E-08	LINC02702
rs499790	11	116649022	C	T	0.1231	0.089841	0.01612	2.77E-08	LINC02702
rs504268	11	116649487	C	T	0.1232	0.089841	0.01612	2.39E-08	LINC02702
rs544479	11	116649681	T	C	0.1231	0.089841	0.01612	2.54E-08	LINC02702
rs506222	11	116649717	A	C	0.1232	0.089841	0.01612	2.51E-08	LINC02702
rs1240773	11	116649810	G	T	0.239	0.067659	0.01233	3.53E-08	LINC02702
rs549125	11	116650209	C	G	0.1282	0.09894	0.01586	4.38E-10	LINC02702
rs533668	11	116650418	T	G	0.1282	0.09894	0.01586	4.39E-10	LINC02702
rs573985	11	116650599	G	A	0.128	0.09894	0.01587	4.14E-10	LINC02702
rs575906	11	116650841	A	G	0.1284	0.098034	0.01585	5.29E-10	LINC02702
rs562756	11	116651258	G	C	0.128	0.097127	0.01587	9.09E-10	LINC02702
rs563796	11	116651394	A	G	0.1291	0.098034	0.0158	5.65E-10	LINC02702
rs563917	11	116651439	C	T	0.1291	0.098034	0.0158	5.90E-10	LINC02702
rs492182	11	116651447	G	A	0.1292	0.098034	0.0158	5.04E-10	LINC02702
rs5795043	11	116651516	TC	T	0.1191	0.091667	0.01648	2.48E-08	LINC02702
rs565607	11	116651613	C	T	0.1291	0.09894	0.0158	3.55E-10	LINC02702

rs567576	11	116651842	A	G	0.1292	0.10075	0.01578	2.01E-10	LINC02702
rs567599	11	116651848	G	T	0.1292	0.10075	0.01578	1.92E-10	LINC02702
rs3017237	11	116651908	G	C	0.1294	0.10075	0.01577	1.90E-10	LINC02702
rs480878	11	116652137	G	A	0.1299	0.10075	0.01575	1.83E-10	LINC02702
rs138535309	11	116652253	CTT	C	0.1293	0.10075	0.01577	1.57E-10	LINC02702
rs202191031	11	116652259	AAGTGG	A	0.1293	0.10075	0.01577	1.57E-10	LINC02702
rs483747	11	116652450	T	C	0.1294	0.10075	0.01576	1.82E-10	LINC02702
rs485638	11	116652656	G	C	0.1294	0.10075	0.01576	1.73E-10	LINC02702
rs528647	11	116653104	C	T	0.1295	0.10075	0.01576	1.57E-10	LINC02702
rs528732	11	116653127	G	A	0.1295	0.10075	0.01576	1.57E-10	LINC02702
rs3017778	11	116653214	C	T	0.1294	0.101654	0.01576	1.32E-10	LINC02702
rs3017779	11	116653271	C	T	0.1294	0.101654	0.01576	1.01E-10	LINC02702
rs480823	11	116655013	T	C	0.1249	0.109751	0.0162	1.07E-11	LINC02702
rs481843	11	116655150	C	T	0.1239	0.107059	0.01627	4.26E-11	LINC02702
rs34485603	11	116655315	CCT	C	0.1238	0.10616	0.01629	6.39E-11	LINC02702
rs486394	11	116655605	A	C	0.1273	0.10616	0.01612	3.89E-11	LINC02702
rs118175510	11	116661831	T	C	0.0896	0.111541	0.01872	2.82E-09	BUD13, LINC02702
rs1787717	11	116682049	C	T	0.3546	-0.08371	0.011	2.64E-14	BUD13, LINC02702
rs5795046	11	116682757	TG	T	0.3545	-0.08425	0.011	1.80E-14	BUD13, LINC02702
rs1787689	11	116697666	A	G	0.4269	-0.07742	0.01069	4.40E-13	BUD13, LINC02702
rs1787691	11	116697997	G	A	0.4263	-0.07775	0.01068	3.31E-13	BUD13, LINC02702
rs71037412	11	116699941	C	CA	0.4291	-0.07548	0.01066	1.44E-12	BUD13, LINC02702

rs1145191	11	116700002	C	G	0.4291	-0.07548	0.01066	1.44E-12	BUD13, LINC02702
rs1145193	11	116700226	A	G	0.43	-0.07645	0.01066	7.40E-13	BUD13, LINC02702
rs11601168	11	116700422	T	A	0.07166	-0.118	0.02015	4.82E-09	BUD13, LINC02702
rs11601584	11	116700568	T	C	0.3606	0.063913	0.01093	3.84E-09	BUD13, LINC02702
rs1145196	11	116701489	C	T	0.4294	-0.07613	0.01066	9.61E-13	BUD13, LINC02702
rs1240783	11	116701809	T	C	0.4293	-0.07602	0.01066	1.00E-12	BUD13, LINC02702
rs4938302	11	116713788	T	C	0.4925	0.057325	0.0105	3.79E-08	BUD13, LINC02702
rs4938303	11	116714271	T	C	0.4926	0.058269	0.0105	3.38E-08	BUD13, LINC02702
rs7350481	11	116715567	C	T	0.2712	0.235072	0.01199	1.24E-85	BUD13, LINC02702
rs7926828	11	116715707	T	C	0.3429	0.162969	0.01116	4.13E-48	BUD13, LINC02702
rs4938304	11	116717709	C	T	0.07185	-0.12988	0.02004	9.12E-11	BUD13, LINC02702
rs79408961	11	116717877	C	T	0.06313	0.216723	0.02214	1.26E-22	BUD13, LINC02702
rs4938305	11	116717913	C	T	0.3417	0.166362	0.01122	6.65E-50	BUD13, LINC02702
rs141882698	11	116730622	A	C	0.08244	-0.1514	0.01893	1.27E-15	BUD13, LINC02702
rs75387300	11	116731683	G	A	0.1491	-0.14283	0.01462	1.56E-22	BUD13, LINC02702
rs4938307	11	116733798	G	A	0.2209	-0.15876	0.01256	1.40E-36	BUD13, LINC02702
rs117170971	11	116734549	G	A	0.08045	-0.1135	0.01912	2.94E-09	BUD13, LINC02702
rs1558860	11	116736652	C	A	0.2327	0.207827	0.0126	2.96E-61	BUD13, LINC02702
rs1558861	11	116736721	T	C	0.2327	0.207014	0.01259	5.99E-61	BUD13, LINC02702
rs9326246	11	116741017	G	C	0.2327	0.20945	0.01261	4.19E-62	BUD13, LINC02702
rs180349	11	116741111	T	A	0.2333	0.20945	0.0126	5.05E-62	BUD13, LINC02702
rs180348	11	116741279	G	A	0.06159	-0.13262	0.02163	8.64E-10	BUD13, LINC02702

rs11216125	11	116741558	G	A	0.2359	-0.15642	0.01225	2.23E-37	BUD13, LINC02702
rs117788500	11	116742318	G	C	0.07645	-0.1216	0.01957	5.08E-10	BUD13, LINC02702
rs17518841	11	116744528	G	A	0.07678	-0.12115	0.01952	5.51E-10	BUD13, LINC02702
rs11216126	11	116746524	A	C	0.2325	-0.1617	0.01228	1.43E-39	BUD13, LINC02702
rs45539536	11	116747179	C	T	0.07694	-0.11957	0.01951	8.91E-10	BUD13, LINC02702
rs57950207	11	116749954	T	C	0.07697	-0.12183	0.0195	4.22E-10	BUD13
rs17440396	11	116751054	G	A	0.07595	-0.12126	0.01963	6.42E-10	BUD13
rs180326	11	116753987	T	G	0.2334	0.215918	0.01259	4.07E-66	BUD13
rs77112220	11	116755510	C	T	0.07681	-0.12059	0.01952	6.37E-10	BUD13
rs17519093	11	116759189	G	A	0.07703	-0.1189	0.0195	1.08E-09	BUD13
rs3825041	11	116760991	C	T	0.2298	0.216723	0.01267	1.75E-65	BUD13
rs139872819	11	116761196	T	C	0.0769	-0.11878	0.0195	1.11E-09	BUD13
rs10488698	11	116763231	G	A	0.07637	-0.11766	0.01957	1.84E-09	BUD13
rs17440824	11	116763457	C	T	0.07882	-0.11766	0.01929	1.06E-09	BUD13
rs1268353	11	116768976	C	T	0.2733	-0.16676	0.01174	7.62E-46	BUD13
rs622604	11	116769346	T	C	0.276	-0.16795	0.01168	7.87E-47	BUD13
rs610675	11	116769378	T	A	0.276	-0.16795	0.01168	7.69E-47	BUD13
rs6589565	11	116769521	G	A	0.2312	0.218332	0.01265	1.29E-66	BUD13
rs623908	11	116769652	A	G	0.2761	-0.16795	0.01168	6.64E-47	BUD13
rs11216135	11	116775346	C	G	0.2736	-0.16901	0.01171	3.22E-47	ZPR1
rs11602073	11	116776142	C	T	0.2737	-0.16617	0.01171	9.94E-46	ZPR1
rs11216136	11	116776404	T	G	0.2759	-0.16735	0.01168	1.54E-46	ZPR1

rs2160669	11	116776891	T	C	0.2325	0.216723	0.01262	4.81E-66	ZPR1
rs964184	11	116778201	C	G	0.2331	0.216723	0.0126	2.36E-66	ZPR1
rs75198898	11	116779090	G	A	0.08822	0.396761	0.01937	2.42E-93	ZPR1
rs113932726	11	116779922	C	T	0.08744	0.400788	0.01946	3.07E-94	ZPR1
rs34248274	11	116779950	CTAGT	C	0.2738	-0.167	0.0117	3.30E-46	ZPR1
rs1942478	11	116780747	T	G	0.2751	-0.16877	0.01168	2.65E-47	ZPR1
rs4417316	11	116781585	C	T	0.2735	-0.16724	0.01169	2.25E-46	ZPR1
rs6589566	11	116781707	A	G	0.233	0.217528	0.01261	1.46E-66	ZPR1
rs7483863	11	116781775	G	A	0.233	0.217528	0.01261	1.46E-66	ZPR1
rs2075290	11	116782580	T	C	0.2495	0.193097	0.01229	1.46E-55	ZPR1
rs603446	11	116783719	C	T	0.2565	-0.1403	0.01194	7.30E-32	ZPR1
rs10750096	11	116786072	A	C	0.2331	0.217528	0.01261	9.89E-67	ZPR1
rs3741298	11	116786845	T	C	0.3904	0.147558	0.01081	2.97E-42	ZPR1
rs3741297	11	116786951	C	T	0.08745	0.408128	0.01947	1.61E-97	ZPR1
rs2072560	11	116791110	C	T	0.2318	0.217528	0.01266	6.43E-66	APOA5
rs662799	11	116792991	A	G	0.323	0.312619	0.01145	7.32E-164	APOA5
rs17120035	11	116793135	C	T	0.07513	-0.12613	0.01979	1.81E-10	APOA5
rs7103224	11	116793250	G	A	0.0751	-0.12624	0.01979	1.79E-10	APOA5
rs7123666	11	116796367	G	A	0.1806	0.19967	0.01408	1.45E-45	APOA5
rs7927820	11	116811440	A	G	0.3298	0.13715	0.01124	2.27E-34	APOA5
rs76636922	11	116812279	G	A	0.3217	-0.06774	0.01125	1.75E-09	APOA5
rs7396835	11	116813312	C	T	0.3303	0.13715	0.01122	3.23E-34	APOA4, APOA5

rs7396851	11	116813448	C	T	0.3301	0.13715	0.01122	1.60E-34	APOA4, APOA5
rs79841374	11	116813844	C	T	0.3209	-0.06806	0.01125	1.45E-09	APOA4, APOA5
rs77535577	11	116814675	G	A	0.3199	-0.06892	0.01129	1.06E-09	APOA4, APOA5
rs5128	11	116832924	C	G	0.3061	0.08526	0.01148	9.84E-14	APOC3
rs10750098	11	116834852	T	G	0.3053	0.083422	0.01151	3.60E-13	APOC3, APOA1
rs61905145	11	116847699	G	A	0.05902	0.198031	0.0341	5.96E-09	SIK3, APOA1-AS
rs888246	11	116853516	C	T	0.05628	0.140631	0.02319	1.26E-09	SIK3
rs12099358	11	116855332	C	A	0.05641	0.138892	0.02316	1.84E-09	SIK3
rs78857232	11	116856749	C	T	0.05612	0.138021	0.02321	2.64E-09	SIK3
rs2080586	11	116862600	T	C	0.05872	0.200489	0.03419	4.28E-09	SIK3
rs11216168	11	116870837	G	A	0.05606	0.13715	0.02321	3.28E-09	SIK3
rs7111854	11	116906110	A	T	0.1129	-0.10048	0.01646	1.03E-09	SIK3
rs7934763	11	116921641	T	G	0.05874	0.201307	0.03425	4.45E-09	SIK3
rs117287138	11	116924163	G	A	0.05869	0.202941	0.03427	3.36E-09	SIK3
rs56314469	11	116924187	G	A	0.05869	0.202941	0.03427	3.36E-09	SIK3
rs61907591	11	116926139	T	A	0.05874	0.201307	0.03425	4.45E-09	SIK3
rs645258	11	116930485	C	A	0.3257	0.062975	0.01129	2.91E-08	SIK3
rs76690043	11	116930813	C	G	0.05874	0.201307	0.03425	4.45E-09	SIK3
rs61907594	11	116931095	C	T	0.05874	0.201307	0.03425	4.45E-09	SIK3
rs79974099	11	116931865	T	A	0.05874	0.201307	0.03425	4.45E-09	SIK3
rs144015791	11	116935158	G	T	0.05874	0.201307	0.03425	4.45E-09	SIK3
rs61624438	11	116939229	C	A	0.05874	0.201307	0.03425	4.45E-09	SIK3

rs12292858	11	116943263	A	C	0.1269	-0.09695	0.01564	5.69E-10	SIK3
rs12421902	11	116943930	A	G	0.08975	-0.10059	0.01819	3.19E-08	SIK3
rs10892049	11	116945500	G	C	0.1269	-0.09706	0.01564	5.48E-10	SIK3
rs7924318	11	116945914	C	G	0.05874	0.201307	0.03425	4.45E-09	SIK3
rs191655881	11	116946939	A	C	0.1269	-0.09706	0.01564	5.48E-10	SIK3
rs4316540	11	116948525	C	T	0.05874	0.201307	0.03425	4.45E-09	SIK3
rs112297132	11	116949116	G	GA	0.05874	0.201307	0.03425	4.45E-09	SIK3
rs61907605	11	116949355	T	A	0.05874	0.201307	0.03425	4.45E-09	SIK3
rs12420518	11	116949372	T	C	0.1269	-0.09706	0.01564	5.48E-10	SIK3
rs1473324	11	116951202	T	G	0.05874	0.201307	0.03425	4.45E-09	SIK3
rs1473325	11	116951318	C	T	0.05874	0.201307	0.03425	4.45E-09	SIK3
rs12286405	11	116951812	C	T	0.1269	-0.09684	0.01564	5.82E-10	SIK3
rs1473326	11	116952956	T	C	0.05874	0.201307	0.03425	4.45E-09	SIK3
rs11216200	11	116961127	C	T	0.1267	-0.09794	0.01566	3.93E-10	SIK3
rs7112967	11	116961196	C	T	0.05874	0.200489	0.03425	4.52E-09	SIK3
rs56134734	11	116962339	G	A	0.05874	0.200489	0.03425	4.52E-09	SIK3
rs61905677	11	116962939	T	G	0.05874	0.200489	0.03425	4.52E-09	SIK3
rs61905678	11	116964069	C	T	0.05874	0.200489	0.03425	4.52E-09	SIK3
rs61905679	11	116964071	C	T	0.05874	0.200489	0.03425	4.52E-09	SIK3
rs61905680	11	116965315	G	A	0.05874	0.200489	0.03425	4.52E-09	SIK3
rs61905681	11	116965493	A	C	0.05874	0.200489	0.03425	4.52E-09	SIK3
rs78044162	11	116966373	C	T	0.1268	-0.09673	0.01566	6.44E-10	SIK3

rs9971422	11	116966657	T	C	0.1263	-0.0964	0.01569	8.27E-10	SIK3
rs11823013	11	116968819	T	C	0.05875	0.202124	0.03425	3.63E-09	SIK3
rs1241657	11	116969045	C	T	0.3245	0.062975	0.01131	2.55E-08	SIK3
rs59294170	11	116970730	A	T	0.05875	0.202941	0.03425	3.34E-09	SIK3
rs17120197	11	116974388	T	C	0.05623	0.142367	0.02322	8.93E-10	SIK3
rs11216210	11	116977613	G	T	0.127	-0.0964	0.01564	7.05E-10	SIK3
rs7109876	11	116977932	C	A	0.05627	0.142367	0.02322	8.13E-10	SIK3
rs7358350	11	116980892	T	C	0.127	-0.0964	0.01564	7.05E-10	SIK3
rs10160754	11	116988278	A	C	0.127	-0.09629	0.01564	7.44E-10	SIK3
rs10160755	11	116988287	A	G	0.127	-0.09629	0.01564	7.44E-10	SIK3
rs61905687	11	116991428	T	C	0.05629	0.142367	0.02321	8.44E-10	SIK3
rs607273	11	116992102	T	A	0.3237	0.063913	0.01127	1.42E-08	SIK3
rs7130531	11	116993117	C	T	0.05629	0.142367	0.02321	8.44E-10	SIK3
rs11216220	11	116993272	T	C	0.1269	-0.09662	0.01564	6.68E-10	SIK3
rs7931665	11	116995524	A	G	0.127	-0.09717	0.01564	5.20E-10	SIK3
rs7950213	11	116997938	C	A	0.05631	0.142367	0.0232	7.90E-10	SIK3
rs10502224	11	116999764	A	G	0.127	-0.09673	0.01564	6.26E-10	SIK3
rs17120233	11	117000507	A	G	0.05631	0.142367	0.0232	7.90E-10	SIK3
rs10892057	11	117001318	A	C	0.1274	-0.0996	0.01564	1.92E-10	SIK3
rs75858236	11	117001547	A	G	0.05631	0.142367	0.0232	8.18E-10	SIK3
rs7946390	11	117004436	A	G	0.127	-0.09695	0.01564	5.65E-10	SIK3
rs11216224	11	117005208	T	C	0.1259	-0.09772	0.01571	5.02E-10	SIK3

rs1940625	11	117005726	A	G	0.127	-0.09695	0.01564	5.68E-10	SIK3
rs11216225	11	117007298	A	G	0.127	-0.09695	0.01564	5.68E-10	SIK3
rs12276805	11	117008043	T	C	0.08951	-0.10192	0.01824	2.29E-08	SIK3
rs12271319	11	117008508	C	T	0.127	-0.09695	0.01564	5.62E-10	SIK3
rs12284696	11	117008803	A	G	0.1261	-0.09684	0.0157	6.89E-10	SIK3
rs7112937	11	117009740	T	C	0.127	-0.09684	0.01564	5.76E-10	SIK3
rs61905691	11	117009987	A	G	0.05631	0.142367	0.0232	8.18E-10	SIK3
rs7125788	11	117012680	T	C	0.05631	0.142367	0.0232	8.04E-10	SIK3
rs61905705	11	117013060	A	T	0.05631	0.142367	0.0232	7.98E-10	SIK3
rs61905706	11	117013061	G	T	0.05631	0.142367	0.0232	7.98E-10	SIK3
rs140947361	11	117013808	AC	A	0.05631	0.142367	0.0232	7.98E-10	SIK3
rs35190172	11	117014597	TC	T	0.05631	0.142367	0.0232	7.98E-10	SIK3
rs17120237	11	117020330	T	C	0.05631	0.142367	0.0232	7.98E-10	SIK3
rs4936359	11	117023008	T	A	0.05631	0.142367	0.0232	7.88E-10	SIK3
rs4936360	11	117023053	G	C	0.05631	0.142367	0.0232	7.87E-10	SIK3
rs111860754	11	117025569	CA	C	0.05631	0.142367	0.0232	7.64E-10	SIK3
rs61905709	11	117027118	C	T	0.05631	0.142367	0.0232	7.87E-10	SIK3
rs4938325	11	117027763	G	A	0.05631	0.142367	0.0232	7.99E-10	SIK3
rs112120205	11	117029070	G	A	0.05631	0.142367	0.0232	7.99E-10	SIK3
rs7112191	11	117031340	G	A	0.05627	0.142367	0.02321	9.55E-10	SIK3
rs4938326	11	117034748	G	C	0.05627	0.142367	0.02321	9.55E-10	SIK3
rs7115242	11	117037567	G	A	0.3248	0.061095	0.01122	4.49E-08	SIK3

rs61905712	11	117038577	G	A	0.05629	0.1415	0.0232	1.11E-09	SIK3
rs112870705	11	117038708	TCAC	T	0.05861	0.198851	0.03429	6.20E-09	SIK3
rs61905713	11	117038713	C	T	0.05861	0.198851	0.03429	6.20E-09	SIK3
rs17120241	11	117044304	T	C	0.05629	0.1415	0.02321	9.86E-10	SIK3
rs76972960	11	117045962	T	C	0.05629	0.1415	0.02321	9.86E-10	SIK3
rs61905716	11	117048351	C	T	0.05629	0.1415	0.02321	9.74E-10	SIK3
rs112151056	11	117048652	G	A	0.05629	0.1415	0.02321	9.74E-10	SIK3
rs61905718	11	117048961	C	T	0.05629	0.1415	0.02321	9.74E-10	SIK3
rs11826651	11	117049977	T	A	0.05629	0.1415	0.0232	1.03E-09	SIK3
rs1815786	11	117050674	C	T	0.3246	0.062035	0.01126	3.49E-08	SIK3
rs61905719	11	117051998	C	T	0.05629	0.1415	0.0232	1.03E-09	SIK3
rs80259223	11	117053602	G	A	0.05702	0.136278	0.02307	3.18E-09	SIK3
rs4938333	11	117060172	A	G	0.05631	0.142367	0.0232	8.83E-10	SIK3
rs61903394	11	117061182	G	A	0.05631	0.142367	0.0232	8.87E-10	SIK3
rs12280210	11	117069733	T	C	0.1246	-0.09728	0.01577	7.08E-10	SIK3
rs9971421	11	117070824	C	T	0.08761	-0.10059	0.01841	4.61E-08	SIK3
rs61903398	11	117070897	C	T	0.05616	0.142367	0.02324	8.63E-10	SIK3
rs202154722	11	117071196	A	AT	0.08885	-0.10026	0.01828	4.17E-08	SIK3
rs17120280	11	117071416	G	A	0.05735	0.136278	0.02302	3.52E-09	SIK3
rs4938336	11	117077726	A	G	0.05887	0.203757	0.03423	2.72E-09	SIK3
rs4936362	11	117081484	C	T	0.0588	0.202124	0.03424	3.52E-09	SIK3
rs61903417	11	117085441	T	G	0.05883	0.202941	0.03423	3.03E-09	SIK3

rs61903418	11	117085949	G	A	0.05883	0.202941	0.03423	3.03E-09	SIK3
rs111901638	11	117089132	G	A	0.05891	0.203757	0.03421	2.73E-09	SIK3
rs11823918	11	117089781	G	C	0.05891	0.202941	0.0342	2.92E-09	SIK3
rs61903421	11	117091086	A	T	0.05891	0.202941	0.0342	2.92E-09	SIK3
rs116861530	11	117091945	G	A	0.08738	-0.10126	0.01843	3.90E-08	SIK3
rs74420345	11	117092561	C	A	0.08737	-0.10148	0.01843	3.66E-08	SIK3
rs7950381	11	117093181	C	A	0.05892	0.202941	0.0342	2.94E-09	SIK3
rs7950501	11	117093249	C	T	0.05892	0.202941	0.0342	2.94E-09	SIK3
rs57383565	11	117093678	A	AG	0.05647	0.139762	0.02318	1.49E-09	SIK3
rs76567973	11	117100045	C	G	0.08838	-0.10292	0.01832	1.91E-08	SIK3
rs78135964	11	117100961	G	T	0.08842	-0.1027	0.01831	2.01E-08	SIK3
rs148233183	11	117101511	G	A	0.08851	-0.10214	0.0183	2.41E-08	SIK3
rs76942203	11	117102531	G	A	0.08851	-0.10225	0.0183	2.31E-08	PAFAH1B2,SIK3
chr11:117103138_A_AAAT	11	117103138	A	AAAT	0.08844	-0.1017	0.01832	2.89E-08	PAFAH1B2,SIK3
rs149576335	11	117104420	G	A	0.08848	-0.10237	0.0183	2.26E-08	PAFAH1B2,SIK3
rs142395187	11	117105293	A	G	0.08785	-0.1037	0.01836	1.64E-08	PAFAH1B2,SIK3
rs17120373	11	117132743	A	G	0.1256	-0.09894	0.01573	3.11E-10	PAFAH1B2,SIK3
rs7934690	11	117140583	T	A	0.06311	0.121332	0.02194	2.89E-08	PAFAH1B2
rs4938355	11	117182959	A	G	0.06248	0.121332	0.02204	3.60E-08	SIDT2
rs73008568	11	117194291	C	G	0.06244	0.122218	0.02205	2.71E-08	SIDT2
rs2075547	11	117197818	C	A	0.06242	0.122218	0.02205	2.78E-08	SIDT2
rs1421085	16	53767042	T	C	0.1337	0.086178	0.01552	2.55E-08	FTO

rs11642015	16	53768582	C	T	0.1332	0.08526	0.01554	4.15E-08	FTO
rs7193144	16	53776774	T	C	0.1308	0.087095	0.01568	2.79E-08	FTO
rs62033400	16	53777876	A	G	0.1302	0.088926	0.0157	1.52E-08	FTO
rs8063057	16	53778521	T	C	0.1302	0.087095	0.0157	2.50E-08	FTO
rs17817449	16	53779455	T	G	0.1296	0.086178	0.01573	4.42E-08	FTO
rs9972653	16	53780451	G	T	0.1301	0.088011	0.0157	2.43E-08	FTO
rs17817497	16	53781523	T	C	0.13	0.088011	0.01571	2.17E-08	FTO
rs8050136	16	53782363	C	A	0.1295	0.086178	0.01573	4.01E-08	FTO
rs8051591	16	53782840	A	G	0.1297	0.086178	0.01572	4.81E-08	FTO
rs9935401	16	53782926	G	A	0.1296	0.086178	0.01572	4.54E-08	FTO
rs3751812	16	53784548	G	T	0.1296	0.086178	0.01573	4.14E-08	FTO
rs3751814	16	53784812	G	A	0.1299	0.088011	0.01572	2.47E-08	FTO
rs9936385	16	53785257	T	C	0.1298	0.087095	0.01572	2.94E-08	FTO
rs9923233	16	53785286	G	C	0.1297	0.088011	0.01573	2.38E-08	FTO
rs11075991	16	53786025	A	T	0.1298	0.087095	0.01572	2.92E-08	FTO
rs11075992	16	53786154	T	C	0.1298	0.087095	0.01572	3.06E-08	FTO
rs9926289	16	53786591	G	A	0.1298	0.087095	0.01572	3.32E-08	FTO
rs9939609	16	53786615	T	A	0.1298	0.087095	0.01572	3.19E-08	FTO
rs7206410	16	53787385	T	C	0.1298	0.087095	0.01572	2.90E-08	FTO
rs7202116	16	53787703	A	G	0.1298	0.086178	0.01571	3.62E-08	FTO
rs7202296	16	53787778	A	G	0.1298	0.087095	0.01572	2.63E-08	FTO
rs66908032	16	53788230	C	A	0.13	0.087095	0.01572	3.16E-08	FTO

chr16:53788257_AT_A	16	53788257	AT	A	0.1297	0.088926	0.01573	1.79E-08	FTO
rs72803697	16	53788271	C	T	0.1298	0.087095	0.01572	3.02E-08	FTO
rs62033403	16	53788325	A	G	0.1298	0.087095	0.01572	2.64E-08	FTO
rs62033404	16	53788327	A	G	0.1298	0.087095	0.01572	2.81E-08	FTO
rs62033405	16	53788475	C	T	0.1296	0.087095	0.01573	3.03E-08	FTO
rs7206122	16	53788528	G	A	0.1298	0.087095	0.01572	2.84E-08	FTO
rs62033408	16	53794050	A	G	0.13	0.087095	0.0157	2.86E-08	FTO
rs17817964	16	53794154	C	T	0.1467	0.082501	0.01491	3.43E-08	FTO
rs173539	16	56954132	C	T	0.215	-0.07526	0.01272	3.25E-09	CETP, HERPUD1
rs247617	16	56956804	C	A	0.1473	-0.14318	0.01463	1.29E-22	CETP, HERPUD1
rs183130	16	56957451	C	T	0.1467	-0.1463	0.01466	1.86E-23	CETP, HERPUD1
rs12149545	16	56959249	G	A	0.1474	-0.14387	0.01463	7.88E-23	CETP, HERPUD1
rs3764261	16	56959412	C	A	0.1489	-0.14341	0.01457	7.16E-23	CETP, HERPUD1
rs36229491	16	56960332	T	TA	0.1498	-0.14318	0.01454	6.85E-23	CETP
rs711752	16	56962299	G	A	0.3876	-0.08676	0.01075	7.08E-16	CETP
rs708272	16	56962376	G	A	0.3875	-0.08654	0.01075	8.68E-16	CETP
rs1864163	16	56963321	G	A	0.1442	0.097127	0.01506	1.23E-10	CETP
rs5817082	16	56963437	C	CA	0.1432	0.097127	0.01512	1.18E-10	CETP
rs12720926	16	56965006	A	G	0.2901	-0.09398	0.01159	5.10E-16	CETP
rs7203984	16	56965346	A	C	0.2857	0.076035	0.01177	1.16E-10	CETP
rs11508026	16	56965416	C	T	0.2872	-0.08818	0.01157	2.51E-14	CETP
rs17231569	16	56965866	CG	C	0.1459	0.09894	0.01503	4.05E-11	CETP

rs12720922	16	56966973	G	A	0.1467	0.10075	0.01501	1.81E-11	CETP
rs4784741	16	56967304	C	T	0.2872	-0.09266	0.01163	1.59E-15	CETP
chr16:56967342_T_TCACA	16	56967342	T	TCACA	0.1452	0.09894	0.01507	4.55E-11	CETP
rs12444012	16	56967526	G	A	0.2888	-0.09124	0.01159	3.47E-15	CETP
rs7205804	16	56970977	G	A	0.302	-0.08414	0.01143	1.83E-13	CETP
rs11076175	16	56972466	A	G	0.133	0.092579	0.0156	3.17E-09	CETP
rs2033254	16	56976073	T	C	0.1408	0.094401	0.01526	6.06E-10	CETP
rs11860407	16	56976916	A	G	0.1406	0.094401	0.01528	5.35E-10	CETP
rs3810307	19	11221894	T	A	0.3539	0.061095	0.01106	3.45E-08	DOCK6
rs12972156	19	44884202	C	G	0.08857	0.132781	0.0188	1.50E-12	NECTIN2
rs12972970	19	44884339	G	A	0.08861	0.132781	0.01879	1.42E-12	NECTIN2
rs34342646	19	44884873	G	A	0.08874	0.132781	0.01877	1.41E-12	NECTIN2
rs6857	19	44888997	C	T	0.09033	0.124869	0.01858	1.97E-11	NECTIN2
rs71352238	19	44891079	T	C	0.09031	0.125751	0.01858	1.23E-11	TOMM40
rs184017	19	44891712	T	G	0.186	0.091667	0.01361	2.02E-11	TOMM40
rs2075650	19	44892362	A	G	0.09054	0.123102	0.01855	2.78E-11	TOMM40
rs34404554	19	44892652	C	G	0.08999	0.125751	0.0186	1.22E-11	TOMM40
rs11556505	19	44892887	C	T	0.0902	0.128393	0.01859	4.93E-12	TOMM40
rs157582	19	44892962	C	T	0.1848	0.096219	0.01364	1.43E-12	TOMM40
rs10414043	19	44912456	G	A	0.09718	0.10436	0.01794	5.88E-09	APOC1
rs7256200	19	44912678	G	T	0.09742	0.105261	0.01794	5.12E-09	APOC1
rs483082	19	44912921	G	T	0.1848	0.091667	0.01366	2.24E-11	APOC1

rs438811	19	44913484	C	T	0.1847	0.090754	0.01367	3.10E-11	APOC1
rs11568822	19	44914381	C	CTTCG	0.1787	0.090754	0.01386	6.65E-11	APOC1
rs5117	19	44915533	T	C	0.1795	0.091667	0.01388	3.36E-11	APOC1

GWAS = genome-wide association study; Mets = metabolic syndrome; SNP = single nucleotide polymorphism; Chr = chromosome; EAF = effect allele frequency; SE: standard error of the beta coefficient.

Supplementary Table S2. Possible mechanisms of candidate genes in metabolic syndrome in the present study.

Gene	Possible mechanisms
CETP	CETP is involved in transferring cholesterol esters and triglycerides between lipoproteins, thereby significantly affecting cholesterol metabolism and lipid balance.
LPL	LPL is crucial in lipoprotein metabolism and the processing of fatty acids. It hydrolyzes triglycerides contained in circulating lipoproteins.
APOA5	APOA5 plays a crucial role in lipid metabolism and is notably associated with increased serum triglyceride levels.
SIK3	SIK3 encodes a protein involved in regulating glucose and lipid metabolism, as well as neuronal development, potentially influencing obesity, metabolic disorders, and neurodegenerative diseases.
BUD13	BUD13 implicates in MetS-related events, notably correlating with elevated serum triglyceride levels.
ZPR1	ZPR1 implicates in MetS-related events, notably correlating with elevated serum triglyceride levels.
APOC1	APOC1 plays a crucial role in lipid metabolism
MLXIPL	MLXIPL is associated with glycerolipid Metabolism.
TOMM40	TOMM40 is linked to fatty acid metabolism.
GCK	GCK is crucial in glucose phosphorylation, and associated with diabetes and metabolic disorders
YKT6	YKT6 is associated with CD8+ T cell levels and under exploration as a potential biomarker for oral squamous cell carcinoma, its relevance to MetS is under investigation.
RPS6KB1	RPS6KB1 is a kinase linked to various cellular processes and protein synthesis, showing associations with conditions like fatty liver disease, intestinal disorders, and oxidative stress pathways.
SENP7	SENP7 is a regulator of SUMOylation, playing roles in maintaining CD8+ T cell metabolic status and contributing to muscle sarcomere organization.
FTO	FTO is related to fatty acid metabolism.
VMP1	FTO is related to fatty acid metabolism.

TUBD1	FTO is related to fatty acid metabolism.
SIDT2	FTO is related to fatty acid metabolism.
SENP7	FTO is related to fatty acid metabolism.
PAFAH1B2	FTO is related to fatty acid metabolism.
DOCK6	FTO is related to fatty acid metabolism.
BCL7B	BCL7B is involved in cell cycle regulation and transcriptional control; its mechanism in metabolic syndrome requires further study.
C19orf80 (ANGPTL8)	C19orf80 (ANGPTL8) is involved in regulating lipoprotein lipase crucial for lipid metabolism, linked to the glycerolipid metabolism pathway.
FOXA2	FOXA2 is related to PPAR Signaling pathways.

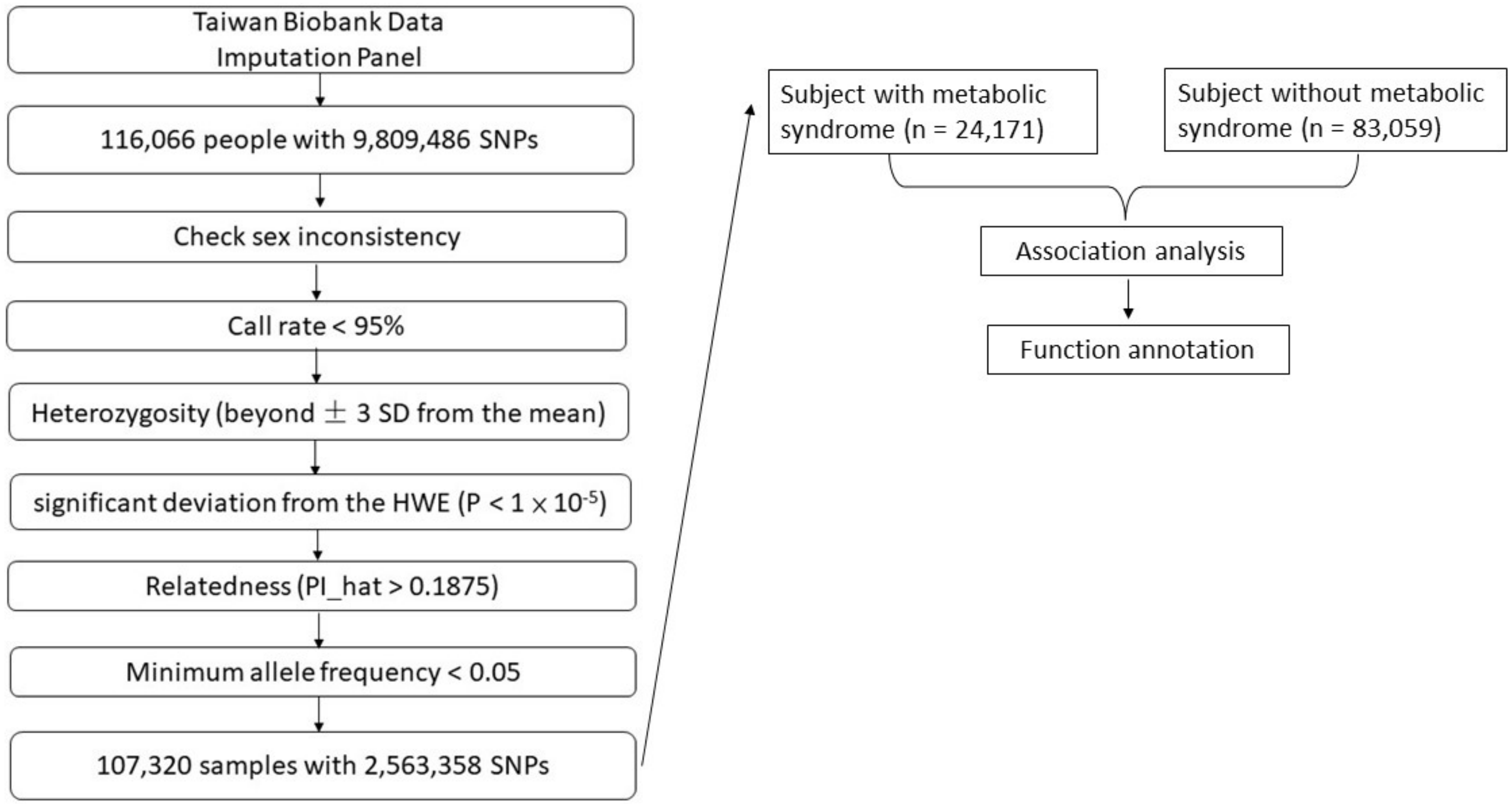

Supplementary Figure S1. The flowchart in this study.
